# ENHANCING COGNITIVE AND EMOTIONAL WELL-BEING THROUGH PIANO TRAINING IN OLDER ADULTS WITH MILD COGNITIVE IMPAIRMENT

**DOI:** 10.1093/geroni/igae098.4343

**Published:** 2024-12-31

**Authors:** Rose Lin, Laura Robinson, Elinor Freer, Kathi Heffner

**Affiliations:** University of Rochester, Rochester, New York, United States; University of Rochester, University of Rochester, New York, United States; University of Rochester, Rochester, New York, United States; University of Rochester, Rochester, New York, United States

## Abstract

Neuropsychiatric symptoms such as depressed mood are increasingly recognized alongside cognitive decline in older adults with mild cognitive impairment (MCI). In response to international guidelines advocating a holistic approach to address both emotional and cognitive symptoms, we conducted a descriptive qualitative study to assess the feasibility and acceptability of a 12-week structured piano training program for seven older adults with MCI. This program included weekly one-hour sessions alternating between individual and group formats, focusing on music theory and scales, with 15-minute daily practices. Content analysis of semi-structured interviews revealed that participants found piano training rewarding, they appreciated integrating piano practice into daily routines, which fostered a sense of purpose and enhanced their ability to manage schedules and handling everyday tasks. Notably, 71.4% of participants enrolled in community school piano class following program completion. They valued interacting with peers facing similar cognitive challenges provided comfort and shared understanding of cognitive decline. Positive reinforcement from instructors and tailored support were crucial in sustaining motivation, particularly when frustrations associated with acquiring new skills, as noted in prior and current studies, could worsen neuropsychiatric symptoms. Recognizing their abilities to learn, despite cognitive decline, boosted self-confidence that often compromised by cognitive impairment. Some adopted piano playing as a new strategy to cope with negative emotions, enhancing overall emotional resilience. Our preliminary findings suggest that piano training has potential to improve well-being in older adults with MCI, setting stage for assessing its long-term impact on cognitive and emotional well-being in larger trials.

